# Investigating the clinical utility of the Eating Beliefs Questionnaire: Validity, reliability and the results of a Confirmatory Factor Analysis

**DOI:** 10.1186/2050-2974-3-S1-O51

**Published:** 2015-11-23

**Authors:** Amy L Burton, Phillipa Hay, Evelyn Smith, Jayanthi Raman, Jessica Swinbourne, Stephen Touyz, Maree J Abbott

**Affiliations:** 1 The University of Sydney, Sydney, Australia; 2 The University of Western Sydney, Sydney, Australia

## 

The Eating Beliefs Questionnaire (EBQ; Groves, Abbott & Baillie, 2015) is a 32-item self-report questionnaire assessing positive and negative beliefs about binge-eating, which are implicated in the maintenance of binge-eating in keeping with Cooper et al.'s (2004) cognitive model. The EBQ was shown to be a valid and reliable measure of eating-related beliefs with excellent psychometric properties. The EBQ has yet to be cross-validated with a clinical sample, and clinical cut-off scores have not been determined. In order to address these issues, a sample of 739 participants was recruited (544 community, 195 clinical: 95 with a diagnosed eating disorder and 100 obese Ps). Participants completed the test-battery, of which 54 clinical participants completed the test-battery before and after receiving psychological treatment for binge-eating, and 38 community participants completed the test-battery again after a lapse of two-weeks. The results of a Confirmatory Factor Analysis will be presented, including evaluation of the EBQ's psychometric properties (internal consistency, criterion validity, construct validity, agreement, reliability, responsiveness, and interpretability). Results will be interpreted with regard to current cognitive models and the EBQ's utility for use in clinical and research settings will also be discussed.

